# Optical pH Sensor Based on a Long-Period Fiber Grating Coated with a Polymeric Layer-by-Layer Electrostatic Self-Assembled Nanofilm

**DOI:** 10.3390/s24051662

**Published:** 2024-03-04

**Authors:** José M. Pereira, João P. Mendes, Bernardo Dias, José M. M. M. de Almeida, Luís C. C. Coelho

**Affiliations:** 1 INESC TEC—Institute for Systems and Computer Engineering, Technology and Science, Rua Dr. Alberto Frias, 4200-465 Porto, Portugal; jmsapereira@outlook.com (J.M.P.); joao.p.mendes@inesctec.pt (J.P.M.); b.m.limposerradossantosdias@uva.nl (B.D.); luis.c.coelho@inesctec.pt (L.C.C.C.); 2 Department of Physics and Astronomy, Faculty of Sciences, University of Porto, Rua do Campo Alegre, 4169-007 Porto, Portugal; 3 School of Sciences and Technology, University of Trás-os-Montes e Alto Douro, Quinta de Prados, 5000-801 Vila Real, Portugal

**Keywords:** long-period fiber grating, optical fiber sensor, pH sensor, polymeric nanocoating

## Abstract

An optical fiber pH sensor based on a long-period fiber grating (LPFG) is reported. Two oppositely charged polymers, polyethylenimine (PEI) and polyacrylic acid (PAA), were alternately deposited on the sensing structure through a layer-by-layer (LbL) electrostatic self-assembly technique. Since the polymers are pH sensitive, their refractive index (RI) varies when the pH of the solution changes due to swelling/deswelling phenomena. The fabricated multilayer coating retained a similar property, enabling its use in pH-sensing applications. The pH of the PAA dipping solution was tuned so that a coated LPFG achieved a pH sensitivity of (6.3 ± 0.2) nm/pH in the 5.92–9.23 pH range. Only two bilayers of PEI/PAA were used as an overlay, which reduces the fabrication time and increases the reproducibility of the sensor, and its reversibility and repeatability were demonstrated by tracking the resonance band position throughout multiple cycles between different pH solutions. With simulation work and experimental results from a low-finesse Fabry–Perot (FP) cavity on a fiber tip, the coating properties were estimated. When saturated at low pH, it has a thickness of 200 nm and 1.53 ± 0.01 RI, expanding up to 310 nm with a 1.35 ± 0.01 RI at higher pH values, mostly due to the structural changes in the PAA.

## 1. Introduction

Most chemical reactions are heavily influenced by pH variations, so the control of this parameter is of high importance to multiple applications such as industrial, biomedical, and environmental [1]. The standard tool for this process is the glass pH electrode [2] since it covers a wide range of pH with high precision and a fast response time, but despite its convenience, there is still a need for other sensing structures to improve the measurement process in specific scenarios such as in batteries, where an electric current flows through the fluids, therefore altering the measurements of electrodes, and inside cells, that require nanosized sensitive structures.

Optical pH sensing is an alternative to glass electrodes, already common through pH indicator stripe tests. They make use of dyes whose absorbance in the visible spectrum changes with this parameter, enabling a fast evaluation of the pH without the need for any device. Recently, optical-fiber-based pH sensors have been receiving more attention due to the need for small sensors for real-time local and in vivo measurements. Several materials were already implemented in multiple sensing schemes [2]. Some sensors made use of the absorbance or luminescence properties of pH indicators [3], therefore being affected by changes in the light intensity and the limited durability of the dyes. Others were coated with pH-sensitive films [4] that change their refractive index (RI) with the pH, usually due to an associated swelling/deswelling phenomenon.

Fiber-optic pH sensors have been investigated in different sensing structures: long-period fiber gratings (LPFGs) [5], fiber Bragg gratings (FBGs) [6], fiber surface plasmon resonance (SPR) [4], and fiber interferometers [7]. Most reported structures are based on pH-sensitive polymers such as polyacrylamide [4], polyaniline [8], poly(methacrylic acid) [9], and polyacrylic acid (PAA) [5]. These polymers are commonly coated on the fiber through dip coating, but since some are polyelectrolytes, by pairing them with another polymer with opposite charge and stacking them alternately, the layer-by-layer (LbL) electrostatic self-assembly technique can be implemented. The foundation of the coating is established upon the principles of electrostatic attraction between polycations and polyanions. This electrostatic interaction facilitates the sequential deposition of alternating layers of each material with precise and controlled thicknesses. The control of the coating’s characteristics is achieved by manipulating various factors during the deposition process, including the concentration of each polymer in solution, their pH, and their exposure time to the substrate during deposition [10].

In this work, the LbL technique was used to deposit a polyethylenimine (PEI) and polyacrylic acid (PAA) film in an LPFG. PAA is a pH-sensitive polyanion, and PEI is a common polycation that, with a tuning of the deposition solutions pH, can be combined to achieve thick films when compared to LbL coatings using other polyelectrolytes [11,12]. This property was exploited to fabricate a pH sensor based on an LPFG coated with only two bilayers, a number significantly lower than other sensors based on polymeric LbL assembled films. Consequently, the fabrication time of the sensor is reduced, which increases the reproducibility of the structure and removes the need for automated fabrication systems. A higher number of bilayers can also reduce the band visibility and increase its width while shifting the band position to wavelengths further away from the optimal operating range of single-mode optical fibers, therefore decreasing the sensor resolution, as opposed to the developed structure. The polymeric coating was characterized by comparing the experimental data from the LPFG to that of a Fabry–Perot (FP) cavity on a fiber tip [13], produced with a similar coating, and to simulation results.

## 2. Materials and Methods

### 2.1. Chemical Reagents

Polyethylenimine aqueous solution (PEI; 50% (*w*/*v*), polyacrylic acid (PAA; Mv 3.000.000), and sodium chloride (NaCl) were purchased from Sigma-Aldrich and were used for the polymeric coatings.

Hydrochloric acid, sodium hydroxide, sodium acetate, glacial acetic acid, sodium phosphate dibasic dihydrate, sodium phosphate monobasic monohydrate, Trizma^®^ Hydrochloride, Trizma^®^ base (≥99.9%), sodium bicarbonate and sodium carbonate were also purchased from Sigma-Aldrich and used to prepare the pH buffers.

### 2.2. Preparation of pH Buffer Solutions

To fabricate and test the polymeric coatings, acetate, phosphate, Trizma^®^, and carbonate–bicarbonate 0.1 M buffer solutions in the 5.00–10.18 pH range were prepared. For each solution, the components and the corresponding amount presented in Table A1 were added to 80 mL of deionized water. After they were dissolved using a magnetic stirrer, their pH was measured and, if needed, adjusted using 0.1 M hydrochloric acid and sodium hydroxide solutions. Water was then added until the buffer volume was 100 mL. The final pH values, presented in Table A1, were measured with the commercial sensor HACH HQ40D at a temperature of 20 °C.

### 2.3. Long-Period Fiber Gratings

Long-period fiber gratings consist of the periodic modulation of the RI on the single-mode optical fiber core. Consequently, light in this region can be coupled from the fundamental core mode to the copropagating higher-order cladding modes, leading to several attenuation bands in the transmission optical spectrum. This coupling only occurs around a specific wavelength for each cladding mode, the resonance wavelength, where the attenuation is maximum. It depends not only on the grating’s physical parameters but also on the external medium RI since it changes the effective RI of the cladding mode, shifting the wavelength position of the corresponding attenuation band. LPFGs can then be used as sensing platforms for pH when coated with pH-sensitive materials whose RI varies with this parameter. A change in the surrounding medium pH will shift the attenuation band and modify its peak value and width.

To determine the effect of a coating with different RI and thickness on the resonance wavelength on the attenuation bands, the simulation of the loss dips was performed. The linearly polarized (LP) propagation modes approximation and the coupled-mode theory were used to obtain the fiber modes and their interaction in a long-period fiber grating. Since there was no need to calculate the entire spectrum, which requires more elaborated algorithms, the modified Bragg condition was used to obtain the resonance wavelength for an LP_vj_ cladding mode [14], which is expressed as follows:

(1) $$\beta_{01}\left(\lambda \right)+s_{0}\zeta_{01,01}\left(\lambda \right)-\left(\beta_{vj}\left(\lambda \right)+s_{0}\zeta_{vj,vj}\left(\lambda \right)\right)=\frac{2\pi }{\Lambda }$$

where $\beta_{01}$ and $\beta_{vj}$ are the propagation constants of the core and cladding modes, respectively, calculated using the transfer-matrix method (TMM) [14,15]; $\zeta_{01,01}$ and $\zeta_{vj,vj}$ are the self-coupling coefficients of the core and cladding modes; $s_{0}$ is the summation of the direct current Fourier components of the RI variation; and Λ is the period of the grating.

The LPFGs were fabricated in standard single-mode fiber with a core diameter of 8.2 μm and a cladding diameter of 1258.2 μm using the induced electric arc technique described elsewhere [16]. The grating period and length of the LPFGs were chosen during fabrication so that the attenuation band of the asymmetric cladding mode LP_16_, corresponding to the 6th order, is in the range from 1500 to 1600 nm with an attenuation around 20 dB, achieved for a grating length of 24 cm.

**Figure 1:**
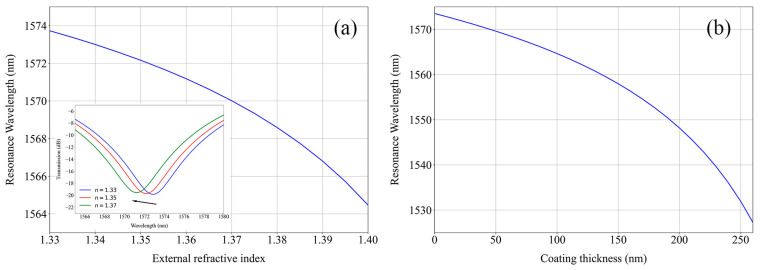
Simulated shift in the LPFG resonance wavelength with a variation in the (**a**) external refractive index (the inset figure presents the shift of simulated resonance bands) and (**b**) thickness of a 1.543 RI coating for a 1.333 external RI.

The fabrication through an electric arc creates a very complex longitudinal and transversal RI profile, and therefore, it is hard to characterize and implement in numerical simulations. Since only the wavelength shift was of interest, for simulation purposes, the fabricated LPFG was approximated to one with a sinusoidal longitudinal RI variation of amplitude 3.6 × 10^−4^ in a sector profile of angles 0° < θ < 270° in both the core and the cladding, as it provides a good description of standard arc-induced gratings with coupling to antisymmetric modes [17]. For a grating period of 395 µm, the response curves in Figure 1 were obtained.

### 2.4. Layer-by-Layer Electrostatic Self-Assembly of the Sensing Coating

The pH-sensitive coating was deposited on top of the LPFG length using the LbL technique by alternating immersion in solutions with the polycation PEI and the polyanion PAA, which are attracted to each other due to electrostatic forces. Since PAA is a weak polyelectrolyte, the properties of the produced film, which include its thickness, depend greatly on the pH of the dipping solution [10]. This parameter also modifies the polymer charge, which consequently affects the adhesion process. When the pH of the PAA dipping solution is low and the pH of the PEI solution is high, the ionization degree of both polymers is low, increasing their diffusion ability and resulting in a higher growth rate of the film. For this reason, the immersion solution of PAA was kept at an acidic level to achieve a higher thickness coating with a low number of bilayers.

**Figure 2:**
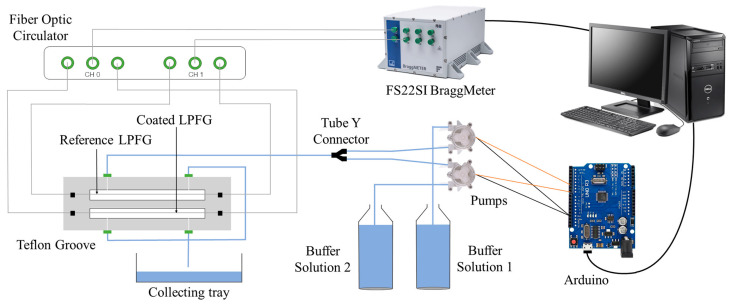
Schematic of the setup for real-time band monitoring during coating fabrication and to obtain the sensor spectral response to pH.

The experimental setup in Figure 2 was used to deposit the pH-sensitive polymeric coating. The sensing region was kept under tension to ensure a constant strain on the LPFG, and the fibers were connected to a BraggMETER (FS22SI from HBK FiberSensing, Portugal, with 1 pm of spectral resolution) using a fiber-optic circulator, which allows for obtaining transmission spectra using a reflection mode interrogator. This unit was connected to a personal computer with developed software that enabled real-time monitoring of the measured spectra, peak tracking, and analysis, with a timeline construction. A vertical lifting platform allowed for easy placement of the sensing region in a Teflon groove so that the LPFG could be immersed in different solutions.

NaCl was dissolved in deionized (DI) water to obtain a 0.5 M solution, in which PEI was diluted to a concentration of 0.1% and pH 10.5. A 0.6% PAA solution was obtained through dissolution in a sodium acetate buffer (pH 5.59), resulting in a final solution with 4.9 pH. Before use, the PEI solution was stirred at room temperature for 2 h, and the PAA was stirred for 8 h.

**Figure 3:**
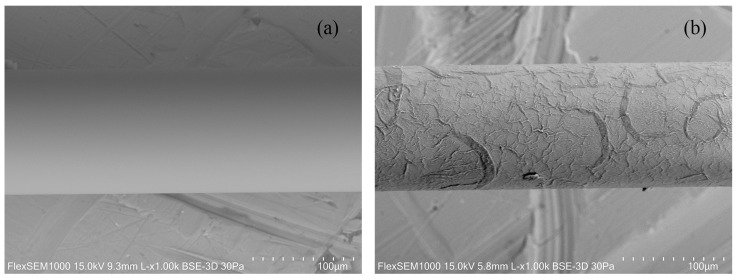
SEM images of (**a**) bare LPFG and (**b**) LPFG coated with 2 PEI/PAA bilayers.

To produce the coating in the LPFG, the fiber was cleaned with acetone, and since the silica fiber surface is negatively charged, it was immersed in the PEI and PAA solutions alternately for 10 min, except for the first PEI monolayer where the immersion lasted 1 h to ensure a homogeneous charge dispersion along the fiber surface. After each layer was assembled, the sensing region was immersed for 1 min in cleaning solutions for PEI and PAA, DI water, and a buffer solution, respectively. This cleaning process was repeated five times to ensure the removal of excess polyelectrolytes between layers. After 2 bilayers, the fiber was left to dry overnight at room temperature. Scanning electron microscope (SEM) images of the fiber before and after this process are shown in Figure 3, confirming the deposition of the polymers.

### 2.5. Sensor Spectral Response to pH

To obtain the response curve of the sensor to pH variations, it was immersed in different pH buffers in the 5.00–10.18 pH range to ensure the solutions’ stability [18]. The curve was obtained using the setup in Figure 2 by sweeping from high pH values to low and the reverse. Another LPFG without the coating was simultaneously immersed in the same buffer solution for temperature and RI compensation.

Multiple successive cycles of exposure of the sensor between 2 different pH buffers were performed using an automatic system, also presented in Figure 2. It consists of a microfluidic chamber, where the LPFGs were placed, connected to two small pumps for the injection of the solutions. The pumps were controlled by an Arduino and an interface on the same software that allowed real-time spectra measurements.

### 2.6. Optical Fiber Interferometer for the Measurement of the Polymeric Coating Refractive Index and Thickness

**Figure 4:**
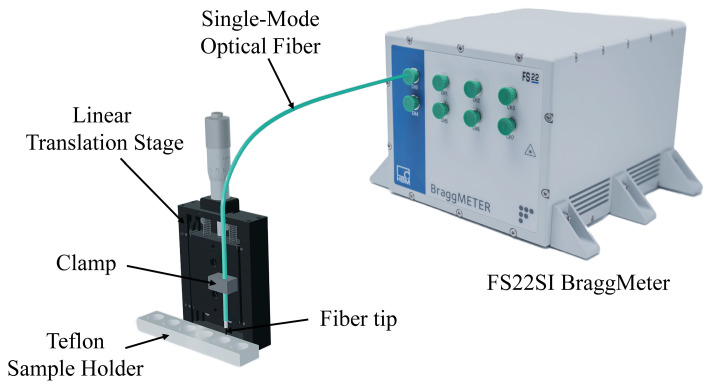
Experimental setup for the fabrication and monitoring of the polymeric coating on a single-mode fiber tip.

Using the experimental setup represented in Figure 4, the procedure used to fabricate the PEI/PAA coating in the LPFG was replicated to deposit 21 bilayers on a single-mode fiber tip. The resulting thickness was enough to produce a low-finesse FP interferometer, whose reflection intensity $I_{R}$ is given by the following equation:

(2) $$I_{R}\left(\lambda \right)=R_{1}+{\left(1-\alpha \right)}^{2}{\left(1-R_{1}\right)}^{2}R_{2}-2\sqrt{R_{1}R_{2}}\left(1-\alpha \right)\left(1-R_{1}\right)cos(\omega )$$

The Fresnel coefficients are $R_{1}={\left(n_{core}-n_{coating}\right)}^{2}/{\left(n_{core}+n_{coating}\right)}^{2}$ and $R_{2}={\left(n_{core}-n_{ext}\right)}^{2}/{\left(n_{core}+n_{ext}\right)}^{2}$, α is the insertion loss factor, and $\omega =4\pi n_{pol}L/\lambda$, where L is the cavity length or the polymeric coating thickness. After a correction of the measured spectra to compensate for the spectral dependency on the intensity of the source, their average value, amplitude, and period can be used to solve Equation (2) for $n_{coating}$, resulting in 4 possible values for the RI and the coating thickness [13]. Therefore, this method provides the ability to characterize the fabricated polymeric coating without the need for previous calibration.

Since a high number of bilayers was deposited and for the early layers deposition there is an exponential growth of the overlay [11,12], this technique cannot be used to obtain the thickness of the LPFG coating, which only has two bilayers but provides relevant information about the expansion/compression behavior of the polymer with the pH as well as its RI.

## 3. Results

**Figure 5:**
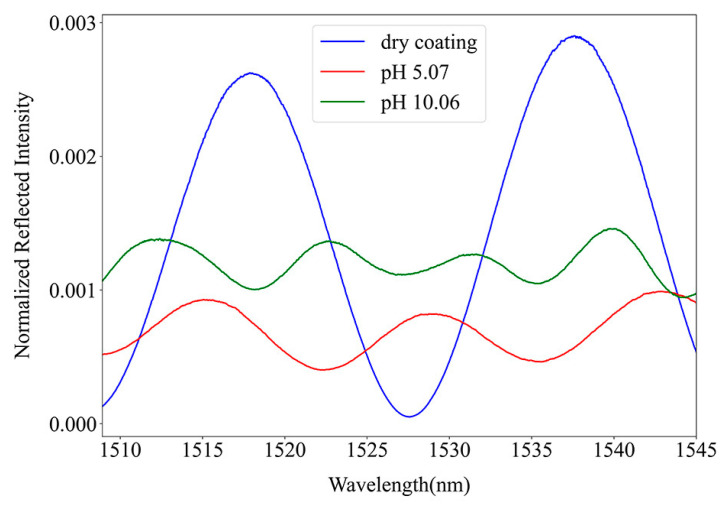
Normalized reflected intensity of an FP cavity in a fiber tip coated with 21 PEI/PAA bilayers.

The spectra of the PEI/PAA coating on a fiber tip obtained at different pH values are represented in Figure 5. It was only possible to obtain those when the structure was dry and at high and low pH values, likely because it was in a saturated state where changes in volume with time were negligible. For this reason, this structure cannot be used as a sensor. Nevertheless, using only the spectra parameters and Equation (2), multiple solutions for the RI of the polymeric coating were obtained, but the difference in the spectra period is large enough to conclude how the film thickness changes with pH. When it is dry, the polymers are in their collapsed state of minimum thickness, and while immersed in a solution, a decrease in the pH results in a compression of the coating, increasing its RI.

**Figure 6:**
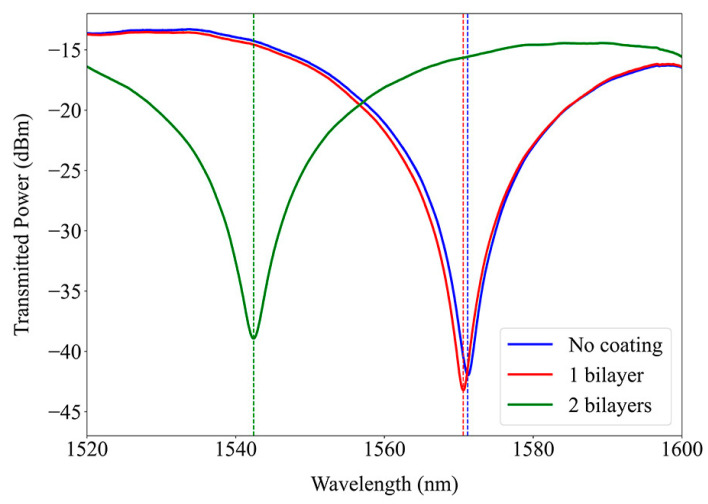
Long-period fiber grating resonance band with the deposition of PEI/PAA bilayers.

During the LbL deposition process, the resonance band was monitored. Figure 6 shows the spectra corresponding to each bilayer when the LPFG was immersed in the sodium acetate rinsing solution. It is evident from the spectra that the bands widen and undergo a blue shift. This observation indicates an increase in the effective RI of the cladding, which is aligned with the expectations since the deposited polymers possess a higher RI than the buffer, therefore confirming the deposition of the overlay. The wavelength shift of the band was significantly higher for the second bilayer due to the exponential growth of the film associated with the diffusion mechanism of the polymers since its chains can penetrate inner layers for charge overcompensation [12].

**Figure 7:**
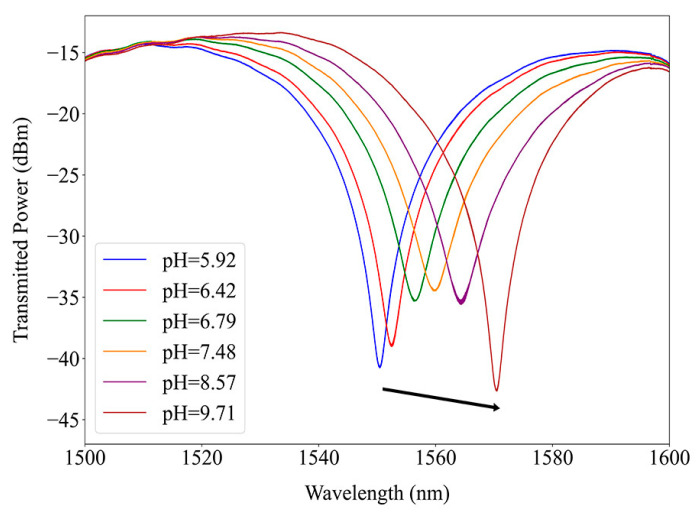
Coated long-period fiber grating resonance bands shift with a pH increase along the arrow.

The coated LPFG spectrum response to an increase in the pH of the immersion solution is presented in Figure 7, where a redshift is observed, confirming the pH sensitivity of the structure. With an increase in the external RI and with higher coating thickness, an LPFG band intensity tends to diminish. This monotone behavior was not observed in the fabricated sensor response to pH, where two regions can be observed, one where the resonance band peak power decreases with the pH and another where it increases. From this result, we can assume that both the variation in the coating thickness and its RI, properties that can oppose each other, influence the sensor response, and neither of those changes can be ignored in the analysis. Since a blueshift with an increase in the pH was recorded, in this situation, the shift due to the coating RI variation dominates over the thickness increase.

**Figure 8:**
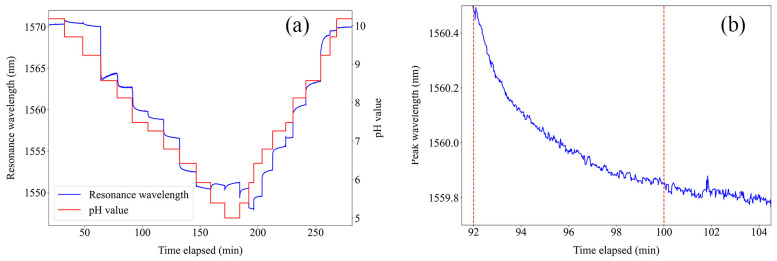
(**a**) Measured dynamic response of the fabricated sensor; (**b**) zoomed-in section to highlight the response time.

The dynamic response of the sensor in the 5.00–10.18 pH range was studied; the results are presented in Figure 8a, where its time response to successive pH variations can be observed. A significant sensitivity to pH was attained in the region from pH 5.92 to 9.23. Outside this range, the sensor exhibited minor wavelength shifts, leading to an absence of any discernible pH response. Figure 8b highlights the sensor behavior when a pH variation is introduced. It takes around 8 min to stabilize, which is significant and, therefore, a factor that must be improved in the sensor to obtain a practical, high-resolution device.

**Figure 9:**
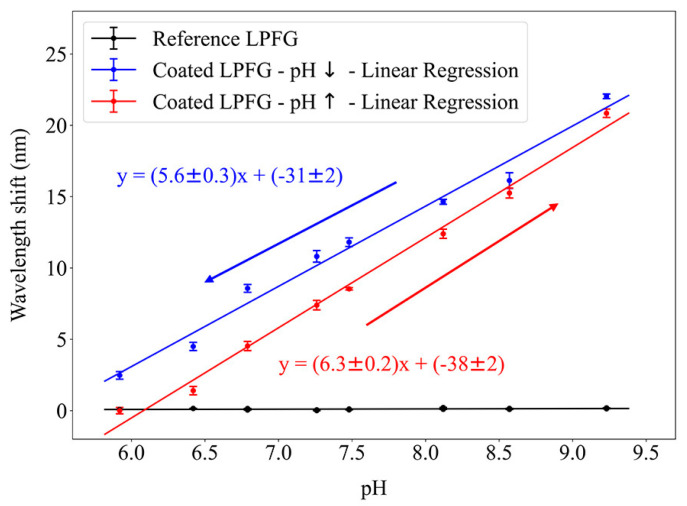
Coated long-period fiber grating response curves for increases and decreases in pH values.

Figure 9 shows the calibration curve of the sensor in the 5.92–9.23 pH range, where the sensitivity to this parameter was registered. When the pH is outside this region, the film is in a stable state, and while certain factors might cause a band shift, it is negligible, and we assume the coating remains in the same state as in the limit of the defined region. The fabricated sensor achieved a substantial wavelength shift of 19.6 nm due to a pH variation of 3.3, while the reference LPFG exhibited a negligible shift. This was obtained in a structure with only two bilayers, whose fabrication was simple and fast despite the critical step of altering the pH of the PAA dipping solution. This can be a disadvantage to the reproducibility of this structure, as environmental factors can affect the solutions’ pH and the LbL deposition process, but since buffer solutions were used, their influence on the overlay thickness and response curve was minimized.

The reversibility of the sensor is also demonstrated, with a 20% hysteresis that cannot be ignored. This behavior is a typical occurrence in pH-sensitive polymers due to differences between the deswelling and swelling phenomena that create non-linear effects. The shrunk and swollen states of the polymeric structure have different energy barrier heights depending on the transition direction. Additionally, the diffusion process of protons when the coating is placed in a new environment varies with the polymer expansion state, resulting in the observed hysteresis and a difference in the response curves when increasing and decreasing the pH [19].

From a linear fit of the experimental data, a sensitivity of (5.6 ± 0.3) nm/pH when decreasing the pH and (6.3 ± 0.2) nm/pH when increasing it was achieved. Higher pH sensitivities could still be obtained by adding additional layers, with the consequence of a wider band.

**Figure 10:**
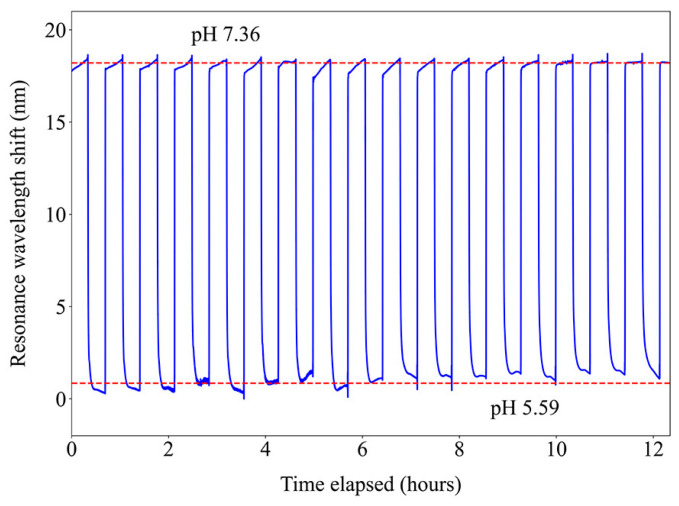
Stability test of the sensor to multiple cycles between pH 5.59 and 7.36.

For this sensor to be practical, reusability and repeatability are essential features. Figure 10 shows the response of the sensor to multiple cycles between buffers of pH 5.59 and 7.36. While a slight degradation of the response was observed, it was negligible considering the overall shift, which is a great result for a possible implementation in long-term measurements. The slight measurement variation might be due to the large and fast variation in pH in the cycles, which can change the number of ionizable groups in the polymers, shifting the response, or due to the formation of salts that alter the ionic strength [19]. The best application of pH-sensitive polymeric coatings is then in real-time monitoring of scenarios where only gradual changes in this parameter are expected.

**Figure 11:**
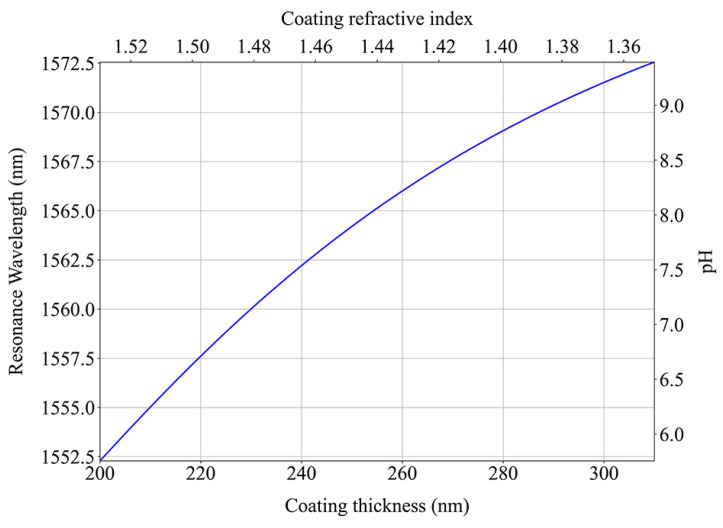
Simulated response of the LP_16_ mode of an LPFG with a variation in its coating thickness and RI (1.333 external RI).

The LPFG sensor achieved a 20 nm wavelength shift of the resonance band when the coating transitioned between saturated states. Additionally, from the FP cavity on the fiber tip, we obtained the possible RI values for the coating at the saturated states and estimated that the film expands up to 55% with the pH increase. With this information, it was possible to infer the coating properties through simulations of the LPFG resonance band displacement to have an approximate idea of the film behavior. The simulated response represented in Figure 11 is similar to the observed experimentally and was achieved when the simulated structure had a coating with an RI of 1.530 and a thickness of 200 nm that expanded to a 310 nm film with a 1.353 RI. For a high pH, the coating is in its expanded state and has a high porosity, and consequently, its RI is close to that of the water. As the pH decreases, the film shrinks, the polymer volume fraction increases, and consequently, the coating RI approaches the value expected for a dry polymer.

From the FP fiber tip experimental data, results show that at a pH of 5.92 (the coating is saturated and compressed), the fabricated overlay has an RI of 1.53 ± 0.01, which expands by (56.6 ± 0.6)% at a pH of 9.23 when its RI is 1.35 ± 0.01.

## 4. Discussion

When the pH value of a multilayer coating changes, there is a competition between multiple factors, such as the electrostatic interaction of polymer chains with themselves or the other polymer chains and the water affinity, resulting in multiple response phases [20]. Since the PEI/PAA coating reduces its thickness with a pH decrease, one can assume that PAA is responsible for most of the structural changes with this parameter since, at low pH, its acidic groups are protonated and un-ionized, the hydrophobic interactions dominate, resulting in a lower thickness, and at a higher pH the opposite occurs, it has a high charge density increasing the electrostatic repulsion between the polymer chains, that associate with water to cause swelling [21].

The reproducibility and replicability of optical sensors remain one of the biggest challenges in this field. Since the coating only has two bilayers, its fabrication time is low, which increases the reproducibility of the LbL technique and, consequently, of the sensor if the pH of the PAA dipping solution is controlled. However, there is still an uncertainty in the sensor fabrication associated with the unpredictability of the electric arc during the LPFG fabrication process. The same fabrication parameters can originate gratings with different RI profiles, lengths, and micro-deformations due to the environmental conditions and the oxidation of the electrodes, resulting in different spectra and, consequently, sensors. Other LPFG fabrication techniques, for example, using UV techniques or a femtosecond laser, might be able to overcome this problem and enable further reproducibility tests. A PEI/PAA coating could also be fabricated on other fiber-optic sensing structures such as Bragg gratings and interferometers, but since they have a different sensitivity to the external medium, the number of bilayers or its thickness would need to be adjusted to achieve a significant pH sensitivity. The possibility of using the absorption properties of the polymers, for example, through sub-wavelength gratings [22], could also be of interest for the development of integrated photonic pH sensors.

The fabricated sensor achieved a 5.6 nm/pH sensitivity over a 5.92–9.23 range, with an 8 min response time. These values are still worse than those achieved by glass pH electrodes, which can measure the entire pH spectrum with resolutions of 0.01 and a response time of a few seconds [23]. Nevertheless, the results are similar to the available commercial optical pH sensors [1], with the additional benefit of using optical fiber technology that enables real-time measurements and multiplexing opportunities. Another pH sensor, based on a smart-hydrogel-coated LPFG, was developed by Mishra et al. [24]. It had a lower sensitivity than the structure developed in this work, 0.66 nm/pH, but it achieved a response time of less than 2 s over a 2–12 pH range, which is favorable for monitoring applications, although no stability tests were made. 

For a practical implementation of the fabricated sensor, its hysteresis and response time still need to be improved, which can be achieved by increasing the overall coating porosity. To do so, the bilayer thickness can be decreased by either increasing the pH of the PAA dipping solution or adding supporting salts to that solution [25]. Both possibilities would result in a decrease in the pH sensitivity, so a compromise must be found between the response time and the sensitivity. Since the LbL technique was used in the fabrication process, it allows the introduction of high RI dielectric materials in between the fiber and the polymeric coating, increasing the LPFG sensitivity [16]. These structures can compensate for the reduced sensitivity of the higher porosity coatings.

## 5. Conclusions

In this work, a pH sensor based on an LPFG coated with two bilayers of PEI/PAA deposited using layer-by-layer electrostatic self-assembly was achieved. A sensitivity of 5.6 nm/pH when decreasing the pH and 6.3 nm/pH when increasing it was obtained in the 5.92–9.23 range. By crossing the information of the sensor response with that of a FP interferometer on a fiber tip and performing simulation work, it can be concluded that the observed changes were due to the coating expanding from a thickness of 200 nm and 1.53 ± 0.01 RI at low pH to 310 nm with a 1.35 ± 0.01 RI at a high value. The reversibility, reusability, and repeatability of the sensor were also demonstrated, but despite having great properties and being easy and fast to fabricate, its hysteresis and response time still need to be improved so it can find a practical implementation.

## Data Availability

Data are available upon request from the authors.

## Appendix A

**Table A1:** Buffer recipes for a volume of 0.1 L and a molarity of 0.1 M.

Buffer	pH *	Component	Amount (mg)
Acetate	5.00	Sodium Acetate Acetic Acid	552.4 196.1
	5.39	Sodium Acetate Acetic Acid	698.9 88.9
	5.59	Sodium Acetate Acetic Acid	772.1 35.3
Phosphate	6.42	Sodium Phosphate Dibasic Dihydrate Sodium Phosphate Monobasic Monohydrate	839.7 947.6
	6.79	Sodium Phosphate Dibasic Dihydrate Sodium Phosphate Monobasic Monohydrate	1312 704.3
	7.26	Sodium Phosphate Dibasic Dihydrate Sodium Phosphate Monobasic Monohydrate	1785 461.0
	7.48	Sodium Phosphate Dibasic Dihydrate Sodium Phosphate Monobasic Monohydrate	2021 339.4
Trizma^®^	8.12	Trizma^®^ Hydrochloride Trizma^®^ Base	788.4 605.4
	8.57	Trizma^®^ Hydrochloride Trizma^®^ Base	406.6 898.9
Carbonate-Bicarbonate	9.23	Sodium Bicarbonate Sodium Carbonate	764.5 95.4
	9.71	Sodium Bicarbonate Sodium Carbonate	528.9 392.6
	10.18	Sodium Bicarbonate Sodium Carbonate	293.4 689.7

* Values measured using the commercial sensor HACH HQ40D at a temperature of 20 °C.
